# Sleep Disorders and Mental Health in Menopausal Women in Tehran

**Published:** 2020-01

**Authors:** Parisa Adimi Naghan, Somayeh Hassani, Makan Sadr, Majid Malekmohammad, Batoul Khoundabi, Javad Setareh, Seyed Mohammad Seyedmehdi, Sharareh Seifi

**Affiliations:** 1 Clinical Tuberculosis and Epidemiologic Research Center, National Research Institute of Tuberculosis and Lung Diseases (NRITLD), Shahid Beheshti University of Medical Sciences, Tehran, Iran,; 2 Virology Research Center, NRITLD, Shahid Beheshti University of Medical Sciences, Tehran, Iran,; 3 Iranian Research Center on Ageing, University of Social Welfare and Rehabilitation Sciences, Tehran, Iran,; 4 Tracheal Diseases Research Center, NRITLD, ShahidBeheshti University of Medical Sciences, Tehran, Iran,; 5 Iran Helal Institute of Applied-Science and Technology, Research Center for Health Management in Mass Gathering, Red Crescent Society of Islamic Republic of Iran, Tehran, Iran,; 6 Psychiatry and Behavioral Sciences Research Center, Addiction Institute, Mazandaran University of Medical Sciences, Sari, Iran,; 7 Chronic Respiratory Diseases Research Center, NRITLD, Shahid Beheshti University of Medical Sciences, Tehran, Iran.

**Keywords:** Menopause, Sleep, Mental health

## Abstract

**Background:**

Sleep complaints are common problems in the general population and insomnia and sleep disorders place significant economic and social burdens on the community. Postmenopausal women are 2.6 to 3.5 times more likely to develop obstructive sleep apnea (OSA) compared to non-menopausal women. In this study, we evaluated sleep disorders and mental health in postmenopausal women.

**Materials and Methods:**

This study was a descriptive cross-sectional study and the samples were selected from postmenopausal women above 50 years who had participated in a survey entitled, “Evaluation of Sleep Disorders among Adults in Tehran” in 2017. Cluster sampling method was applied with proportional allocation. A total of 4021 samples were collected, 2075 of which belonged to women. In addition, 174 out of 2075 samples were related to postmenopausal women over the age of 50. The data were analyzed using the statistical package IBM SPSS version 22.0. P-values less than 0.05 were considered significant.

**Results:**

In this study, 118 (67.8%) women had insomnia for less than three months, and 23 (13.2%) women had insomnia for more than three months. The prevalence of STOPBANG parameters in this group of postmenopausal women was 37% and significantly related to Body mass index (BMI) and neck circumference at P < 0.001 and 0.006, respectively. There was no significant relationship between social dysfunction and insomnia. However, anxiety in General Health Questionnaire (GHQ) was significantly associated with insomnia, sleepiness, sadness, and irritability.

**Conclusion:**

Our results indicate that the impact of insomnia symptoms, OSA comorbidity and mental disorders could extend far beyond. The use of urgent health care and quality of life issues is essential for long-term mental and physical well-being; if there is no treatment in the menopause population, there will be serious mental and physical complications.

## INTRODUCTION

Sleep complaints are common problems in the general population. In fact, about 35% of population has difficulty falling asleep, staying asleep, or waking up early and feel tired even after sleep (1). As age advances, the quality and quantity of sleep decrease due to a reduction in the non-rapid eye movement (NREM) sleep (N3 stage or delta-wave sleep) and rapid eye movement (REM) sleep and also because of increase in sleep disorders (2).

Sleep disorders in women are twice as common as men (2–4). Although deterioration of sleep quality with age in women may be associated with the aging process, the results of various studies have shown that menopause has an independent effect on sleep problems in this population, regardless of age (5, 6). Menopause refers to the permanent cessation of menstruation due to the reduced activity of female sex hormones following the reduced function of ovarian follicles (3, 7).

Generally, sex hormones, especially estrogen, play an important role in women’s health and sleep quality (8,9). Additionally, the quality of sleep during post menopause is negatively influenced by the individual’s poor perception of her health status, poor sleep quality, anxiety, moodiness, chronic diseases, changes in body temperature, hot flashes, circadian rhythm disorders, increased stress responses, behavioral changes, cultural and racial factors, and even ethnic background (5, 10, 11).

Studies on the symptoms of postmenopausal women have shown that 20–60% of these women have sleep-related complaints (8), especially obstructive sleep apnea (OSA) (12). The prevalence of insomnia increases from 38% in premenopausal women to 46–48% in postmenopausal women (2). Meanwhile, OSA seems to be more common in men than women. However, the results of different studies have shown that the prevalence and severity of OSA increases during menopause (13).

According to statistics, postmenopausal women are 2.6 to 3.5 times more likely to develop OSA compared to non-menopausal women (2, 6). In addition, weight gain and reduction in estrogen and progesterone levels during menopause increase the risk of OSA (13). Although both reduction and/or cessation of female sex hormones play an important role in the pathogenesis of OSA, the pathophysiology of their effects remains unknown (14).

Generally, insomnia and sleep disorders place significant economic and social burdens on the community due to reduced productivity, increased risk of accidents, and increased healthcare costs (1, 15). Clearly, insomnia reduces an individual’s quality of life by reducing their concentration and motivation and increasing daytime fatigue, physical and mental burnout, irritability, and disturbance in interpersonal relationships (10). In addition, various studies have shown that people with poor sleep quality are prone to chronic diseases, such as cardiovascular diseases and diabetes mellitus (16, 17). Also, poor sleep quality can increase the risk of falling and decrease mobility and independence among people at older age (15).

With advances in medical sciences and increased life expectancy in recent years, many women are expected to reach the age of menopause in near future. The number of postmenopausal women is speculated to reach 1 200 000 000 by 2030, with developing countries expecting a more dramatic increase (18). If the average life expectancy of women is considered to be 80 years, women spend about one third or more than one third of their lives in the postmenopausal stage. Therefore, timely diagnosis and treatment of sleep problems can be effective in improving the quality of life of women in this age group (12).

In view of the fact that limited studies have been conducted in Iran on factors affecting insomnia and its different types, in this study, we aimed to determine the prevalence of OSA, insomnia, mental complications, and related factors among postmenopausal women to promote future studies in this area and improve women’s quality of life during menopause.

## MATERIALS AND METHODS

In this descriptive cross-sectional study, the samples were selected from postmenopausal women above the age of 50, who had participated in a survey entitled, “Evaluation of Sleep Disorders among Adults in Tehran” in 2017. This study was approved by the ethics committee of Shahid Beheshti University of Medical Sciences.

In this study, cluster sampling method was applied with proportional allocation. A total of 105 clusters were selected from the urban districts of Tehran. Then, the samples were randomly allocated to each class in the clusters. In each cluster, one household was randomly selected as the head of the cluster, and then, ten nearby clusters were systematically selected in a clockwise manner. Finally, in each household, one man and one woman over the age of 18 years were surveyed. A total of 4021 samples were collected, 2075 of which belonged to women. In addition, 174 out of 2075 samples were related to postmenopausal women over the age of 50. Menopause was defined as the absence of menstruation for at least 12 months.

Data were collected using a questionnaire consisting three parts. The first part included data such as snoring, tiredness, obstruction during sleep, hypertension, Body mass index (BMI), neck circumference, age, and gender (STOPBANG). If three or more STOPBANG parameters turn out to be positive, STOPBANG would be considered a positive one. Level of education, history of menopause, and diabetes mellitus were also incorporated within questionnaire items.

Second part “Insomnia Screening Questionnaire (ISQ)” consisted of 18 items with omission of two overlapping questions that were in common with STOPBANG questionnaire. Each item is rated as 1-“never or almost never”; 2-“once or twice a month”; 3-“once or twice a week”; 4-“three or four times a week”; 5-“almost every day”. Reliability of ISQ was assessed by calculating Alpha Cronbach which was 0.79. Subsequently, all items were investigated and the answers were scored.

The third part of the questionnaire was the Iranian version of the 12-item General Health Questionnaire (GHQ-12). GHQ-12 is used to assess the general health of the adults and detect the subjects at risk of non-psychotic disorders. Individuals with GHQ-12 scores of 5 or higher indicated a “poor mental health status”.

### Statistical Analyses

The data were analyzed using the statistical package IBM SPSS version 22.0 (Statistical Package for the Social Sciences, Chicago, IL). The categorical variables are expressed as proportions and frequencies. The continuous variables are summarized as mean±SD. Also, in order to explore the independent nature of some categorical variables, Chi-square was used. P -values less than 0.05 were considered significant. Comparing the average of Chronic Insomnia group with the other two groups was done via running CW.

### Ethical Issues

The study was approved by the Ethics Committee of National Research Institute of Tuberculosis and Lung Diseases. In addition, oral consent was obtained from all participants before enrolling in the study.

## RESULTS

The study included 174 postmenopausal women over the age of 50. The information on weight, height, neck circumference, age, education, and BMI along with other information related to diseases and marital status is presented in Table 1. Of 174 postmenopausal women, 33 (19%) had no sleep problems. On the other hand, 118 (67.8%) women had insomnia for less than three months, while 23 (13.2%) women had insomnia for more than three months. It seems psychiatric disorders and restless leg syndrome might be the most important causes of insomnia as 61% and 29%, respectively (Table 2).

**Table 1.** Demographic Data of Postmenopausal Women

	Mean ± SD
Weight (kg)	70.9± 11.7
Height (cm)	156.7 ± 6.6
Neck circumference (cm)	37 ±3.3
Age (year)	60.4 ± 8.8
Education (years)	8.7± 5
Body mass index (BMI)	27.4± 4.9
Diabetes mellitus	24.7%(43)
Blood pressure	34.5%(60)
Heart disease	23.6 %(41)
Marital status	
Single	5.7%(10)
Married	93.7%(163)
Other	0.6%(1)

**Table 2.** Possible Causes of Insomnia in the Postmenopausal Women (ISQ Questionnaire)

**Questions**	**Chronic insomnia**	**Short sleep insomnia**	**Without Insomnia**	**p-value**
Do you take anything to help you sleep or consume alcohol or drugs?	54.5%^*^	16.2%	3%	0.001<
Do you have any medical conditions that disrupt your sleep?	59.1%^*^	37.6%	12.1%	0.001
Have you lost interest in hobbies or activities?	61.9%^**^	45.3%	28.1%	0.051
Do you feel sad, irritable, or hopeless?	73.9%^*^	61%	30.3%	0.001<
Do you think something is wrong with your body?	65.2%^*^	27.2%	18.2%	0.001<
Are you a shift worker or is your sleep schedule irregular?	52.2%^*^	23.7%	6.1%	0.001<
Are your legs restless and/or uncomfortable before bed?	56.5%^*^	36.4%	18.2%	0.013
Have you been told that you are restless or that you kick your leg in your sleep?	13%	4.2%	3%	0.414
Do you have any unusual behaviors or movements during sleep?	0	0	0	0

* Significant level of 0.05

** Significant level 0.1

The STOPBANG parameters in menopausal women over 50 turned out to be 30.7% on average and found to be significantly related to BMI and neck circumference at P < 0.001 and 0.006, respectively. Other factors such as educational level and marital status in postmenopausal women had no significant relationship with sleep problems. It is noteworthy that the highest percentage of STOPBANG positive (66.7%) was found in age category of 65–69, and interestingly, the percentage was increasing up to this age category and decreasing beyond it (Table 3,4).

**Table 3.** Age Distribution of Postmenopausal Women with Moderate to Severe Obstructive STOPBANG

**Age category (year)**	**Total number of postmenopausal women**	**Number of postmenopausal women with STOPBANG positive (%)**
**50–54**	56	55.6%(31)
**55–59**	29	48.3%(14)
**60–64**	39	53.8%(20)
**65–69**	21	66.7%(14)
**70–74**	17	41.2%(7)
**75**	12	27.3%(3)
**total**	174	30.7%(53)

**Table 4.** Status of Insomnia Effective Factors in Menopausal Women Given the STOPBANG Status

**Questions**	**STOPBANG−**	**STOPBANG +**	**P-Value**
Do you take anything to help you sleep or consume alcohol or drugs?	16.9(14)	30.8(28)	0.035
Do you have any medical conditions that disrupt your sleep?	9.8(8)	26.7(24)	0.006
Have you lost interest in hobbies or activities?	12.3(10)	25.8(23)	0.024
Do you feel sad, irritable, or hopeless?	16.9(14)	39.6(36)	0.001
Do you think something is wrong with your body?	19.3(16)	40.7(37)	0.05
Are you a shift worker or is your sleep schedule irregular?	14.5(12)	33(30)	0.005
Are your legs restless and/or uncomfortable before Bed?	25.3(21)	45.1(41)	0.007
Have you been told that you are restless or that you kick your legs in your sleep?	3.6(9)	6.6(6)	0.587
Do you have any unusual behaviors or movements during sleep?	0(0)	6.6(5)	-

### General Health Quality (GHQ) in Postmenopausal Women

**Figure 1.**
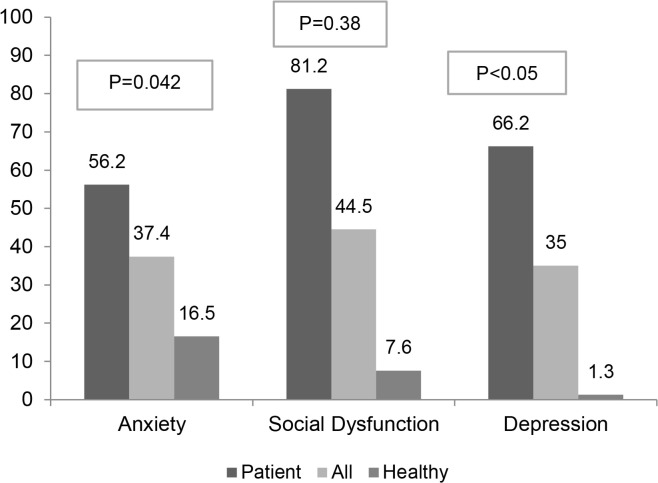
Detailed comparison between GHQ disorders based on three factors in three namely healthy, patient, and all populations

In postmenopausal women, the cut-off point of GHQ questionnaire for discrimination of healthy and unhealthy populations is ≥ 5; in other words, a score of five or higher indicates a problem, while a score less than five indicates a normal status. Based on the findings, 49.7% of menopausal women suffered from mental disorders. Considering the overlap of 12 questions, they were classified into three categories according to the psychiatrists’ expert opinion after examination and validation. These three categories were anxiety 56.2%, social dysfunction 81.2%, and depression 66.2%, respectively. There was no significant relationship between social dysfunction and insomnia (Figure 1). However, anxiety in GHQ was significantly associated with insomnia, sleepiness, sadness, and irritability. Anxiety in GHQ was significantly correlated with insomnia for which alcohol and drugs were consumed (P-value<0.05). Although for other questions in ISQ in table 2 no significant correlation was observed.

## DISCUSSION

The general objective of this research was to survey sleep disorders and their mental consequences on postmenopausal woman using a relatively short questionnaire.

In this study, the risk of moderate to severe obstructive sleep apnea was 30.7% in postmenopausal women based on STOPBANG questionnaire. Also, women in the age range of 65–69 years were the most vulnerable group to obstructive sleep apnea (66.7%). Given the fact that this figure 1 is derived from the STOPBANG screening questionnaire, and according to previous studies (19), at least half of these individuals in the PSG are prone to moderate to severe OSA, accordingly about 15% of the patients in this group are affected (19,20). These results are consistent with community-based SWAN (The Study of Women’s Health Across the Nation) which as a multi-site, longitudinal, and epidemiologic research project aiming at examining the health of women during their middle age (21). OSA is the most common type of sleep apnea, which is caused by recurrent episodes of respiratory arrest, resulting from complete or partial obstruction of the upper airways (14). The decrement in estrogen and progesterone during post-menopause likely plays a role in increased risk for OSA; progesterone enhances ventilatory drive and affects dilatory muscles of pharynx (22) and partly mediates these effects through estrogen-dependent receptors (23); hence, the menopausal decline in both these hormones might impact respiration.

The onset of menopause, which is followed by an increase in age and BMI, increases the risk of OSA. According to multiple studies, OSA is more common in postmenopausal women than non-menopausal women, even after controlling BMI and age (24). Agan et al. estimated the prevalence of moderate to severe OSA and found it to be 45.5%, based on polysomnography (7). In another study by Jehan et al., the prevalence of OSA was estimated at 47–67% after menopause (23, 25). OSA leads to oxidative stress, inflammatory reactions, endothelial damage, sympathetic activity, and metabolic disorders and can predispose a person to arteriosclerosis, and systemic hypertension at this age range (23, 25). Considering these consequences, these people are prone to myocardial infarction and cerebrovascular accident.

In this study, insomnia of more than 3 months is considered remarkable; it is about 13.2%. 54 percent of these patients use alcohol and drugs to relieve insomnia which is an indication of the severity of the disorders in these patients (Table 2). It seems circadian rhythm disorders, psychiatric disorders and **Restless leg syndrome** (RLS) according to ISQ questionnaire might be the most important causes of insomnia in postmenopausal women. Limited data also suggest that differences in sleep-wake cycle regulation (advanced circadian phase) could contribute to sleep difficulties (26), particularly a more fragmented sleep or early morning awakening, in postmenopausal women. Considering all pieces of evidence, special attention needs to be paid to non-hormonal strategies. They are as important as hot flash and other autonomic dysfunction in menopause. Combination treatments, including cognitive behavioral therapy for insomnia and hormonal and non-hormonal pharmacological options are of great significance. (27)

Therapy in order to get immediate benefits as well as enjoy advantages of maintaining optimal health in the postmenopausal years can be achieved by this approach (28).

About half of the patients suffered from mental disorders especially anxiety, depression and poor social performance. There seems to be a remarkable percentage of anxiety in Iranian middle-age women’s population which in its turn may also be effective in insomnia of menopausal individuals and vice versa. The association between menopausal status and psychological distress highlights the need for organizing specific mental health services for middle aged women particularly in relation to menopause (28, 29).There are several limitations to our study. First, OSA was assessed based on STOPBANG questionnaire not polysomnography. Next, another limitation of this study is the lack of access to the patients’ autonomic symptoms.

## CONCLUSION

Our results indicate that the impact of insomnia symptoms, OSA comorbidity and mental disorders could extend far beyond. Receiving urgent health care and maintaining quality of life are essential to long-term mental and physical well-being; if there is no treatment in the menopause population, there will be serious mental and physical complications.
